# Mechanistic insights into the effect of humidity on airborne influenza virus survival, transmission and incidence

**DOI:** 10.1098/rsif.2018.0298

**Published:** 2019-01-16

**Authors:** Linsey C. Marr, Julian W. Tang, Jennifer Van Mullekom, Seema S. Lakdawala

**Affiliations:** 1 Civil and Environmental Engineering, Virginia Tech, Blacksburg, VA 24061, USA; 2 Clinical Microbiology, University Hospitals Leicester NHS Trust, Leicester, UK; 3 Infection, Immunity and Inflammation, University of Leicester, Leicester, UK; 4 Statistics, Virginia Tech, Blacksburg, VA 24061, USA; 5 Microbiology and Molecular Genetics, University of Pittsburgh School of Medicine, Pittsburgh, PA 15219, USA

**Keywords:** humidity, temperature, environment, transmission, flu, aerosol

## Abstract

Influenza incidence and seasonality, along with virus survival and transmission, appear to depend at least partly on humidity, and recent studies have suggested that absolute humidity (AH) is more important than relative humidity (RH) in modulating observed patterns. In this perspective article, we re-evaluate studies of influenza virus survival in aerosols, transmission in animal models and influenza incidence to show that the combination of temperature and RH is equally valid as AH as a predictor. Collinearity must be considered, as higher levels of AH are only possible at higher temperatures, where it is well established that virus decay is more rapid. In studies of incidence that employ meteorological data, outdoor AH may be serving as a proxy for indoor RH in temperate regions during the wintertime heating season. Finally, we present a mechanistic explanation based on droplet evaporation and its impact on droplet physics and chemistry for why RH is more likely than AH to modulate virus survival and transmission.

## 1. Introduction

There has been a surge of interest in understanding transmission and seasonality of influenza and other infectious diseases. Humidity, among other factors, appears to play an important role in influenza transmission, and a confluence of recent studies has suggested that absolute humidity (AH) rather than relative humidity (RH) modulates influenza virus survival, transmission and seasonality [1–4]. While we have an intuitive understanding of humidity in general, the relationship between RH and AH is less obvious. AH is a mass concentration that describes the amount of water vapour per volume of air. Air contains water vapour at concentrations ranging from near-zero grams of water vapour per cubic metre of air in the driest deserts to approximately 40 g m^−3^ in hot and humid environments. The amount of water vapour in air can also be expressed as a partial pressure in units such as atmospheres or millibars. A related concept is specific humidity, which is the mass of water vapour per mass of air, such that absolute and specific humidity are related by the density of air.

RH is defined as the ratio of the actual water vapour pressure (or concentration) to the saturation vapour pressure (or concentration), which is defined as the equilibrium partial pressure of water vapour above a flat surface. The saturation vapour pressure increases with temperature, so the concentration of water vapour can be higher in warmer air. The relationship between AH and RH depends on temperature. At 25°C and 100% RH, the corresponding AH is 23 g m^−3^, while at 0°C and 100% RH, the corresponding AH is 4.8 g m^−3^. On the other hand, an AH of 4 g m^−3^ corresponds to an RH of 83% at 0°C and 17% at 25°C. Greater detail on different measures of atmospheric moisture and their relationship to human health can be found in Davis *et al*. [5] and Wolkoff [6].

Although recent studies suggest that AH modulates influenza virus survival, transmission and seasonality [1–4], we contend that the combination of RH and temperature provides a more consistent, mechanistically sound explanation for the observations. To support our contention, we review and reanalyse previously published data while adding mechanistic insights to the findings in this perspective article. In evaluating studies of influenza virus survival in aerosols and droplets, transmission in animal models and influenza incidence, we make two critical observations. First, there is strong evidence that temperature modulates influenza virus survival independent of humidity [7]; survival is reduced at higher temperatures. Higher levels of AH are physically possible only at higher temperatures, and because of this relationship, directly comparing the effect of AH versus RH alone on virus survival paints an incomplete picture. Second, most influenza transmission between humans probably takes place indoors within a narrow range of temperatures, where AH and RH move in tandem. When the outdoor temperature is low and the indoor environment is heated, indoor RH is closely correlated with outdoor AH but is only weakly correlated with outdoor RH. Thus, we expect outdoor AH, rather than outdoor RH, to be a better predictor of influenza incidence. Finally, we propose a mechanistic explanation for the relationship between RH and transmission via droplets and aerosols, where RH affects their physics and chemistry.

## 2. Material and methods

### 2.1. Statistical analyses

To investigate the relationships between the dependent variable of virus viability (quantified as log % viability) and independent variables of temperature (*T*), AH or RH, we fitted statistical models in JMP^®^ Pro, v. 13 using ordinary least-squares regression with normal error structure. We used techniques consistent with best statistical practices in regression modelling, including considering a variety of metrics of fit and prediction, using plots to inform modelling and removing collinear terms. We evaluated models on the basis of *F* and *t*-tests for statistical significance, *R*^2^, mean squared error, residual plots, variance inflation factors and predicted versus actual plots.

In selecting among predictive statistical models for virus viability, we must account for the relationship between AH and RH. Based on an improved form of the Magnus equation for the saturation vapour pressure of water [8], the ideal gas law and the definitions of AH and RH, we derived the following empirical equation for AH as a function of RH and temperature (*T*):

2.1 $$\mathrm{AH}=\frac{1322.7\exp(17.625\;T/(T+243.04))\times (\mathrm{RH}/100)}{T+273.15},$$

where AH is in g m^−3^, *T* is in °C and RH is in per cent. This equation is valid over the temperature range of −40 to 50°C. Further details of the derivation appear in the electronic supplementary material.

### 2.2. Equilibrium droplet size

We followed a parametrization for the relationship between RH and droplet size presented in a study of the interaction of aerosols composed of salt and protein with water vapour (eqn 28 in [9]). The equation assumes that the solutes in the droplet do not interact with each other and that the volume of the droplet is the sum of the volume of the dry solutes and of the water. We represented salt by NaCl at a concentration of 9 mg ml^−1^ protein as having a molecular weight of 640 000 g mol^−1^ and a concentration of either 3 mg ml^−1^ as found in nasal airway surface fluid [10] or 76 mg ml^−1^ [11] as estimated for aerosols of respiratory breath condensate, and surfactant by dipalmitoylphosphatidylcholine (DPPC) with a molecular weight of 734 g mol^−1^ and a concentration of 0.5 g l^−1^ [12]. We applied the simplified ion-interaction parametrization of the osmotic coefficient (*Φ*) for the strong electrolyte NaCl (eqn 13 in [9]) and the osmotic pressure parametrization of *Φ* for protein and surfactant, assumed to behave like rigid spheres. We assumed that the surface tension of the solution equalled that of water; the predicted diameter was not sensitive to this value (i.e. if it were a factor of three lower, as in the lungs) for the size of droplets under consideration.

## 3. Results and discussion

### 3.1. Prior study of airborne virus survival

To our knowledge, only one study has examined airborne influenza virus survival as a function of both temperature and humidity. Harper [13] used a Collison nebulizer to aerosolize virus from a suspension in ‘casein McIlvaine's buffer’ into a 75-l rotating drum. He measured viability in terms of infectious dose in egg membranes, corrected for physical loss, at three temperatures (7.0–8.0°C, 20.5–24.0°C and 32.0°C) and five RHs (20–25%, 34–36%, 49–51%, 64–65% and 81–82%). His results showed that the virus survived better at the lowest temperature and the lowest RH tested. Shaman & Kohn [3] reanalysed Harper's results and showed that AH, in terms of the vapour pressure of water, provided a much stronger fit to virus viability than did RH.

### 3.2. Effect of temperature and humidity on virus survival

A systematic review of influenza virus persistence concluded that temperature, over an environmentally relevant range, is a significant predictor of persistence across all environmental matrices considered, including air, water and soil [7]; higher temperature is associated with a shorter half-life. The effect of temperature on influenza viruses has also been studied in *in vitro* culture systems in the context of live virus vaccine attenuation. A serial passage of viruses at restricted temperatures produced viruses with temperature sensitivity (*ts*), such as influenza viruses with restricted growth at 39°C, or a cold-adaptive (*ca*) phenotype, whereby virus growth was enhanced at 33°C compared with 37°C [14,15]. However, these studies on live vaccine platforms focused on the growth restriction and not the survival of virus strains at various temperatures.

Researchers have proposed mechanisms for the thermal degradation of influenza virus, and we expect these to apply across a wide variety of systems, such as aerosols, droplets, surfaces or bulk culture medium. Woese [16] identified thermal denaturation of proteins and nucleic acids as mechanisms of virus inactivation and invoked the Eyring equation, used to describe the rate of chemical reactions and functionally similar to the Arrhenius equation, to explain temperature dependence. Polozov *et al*. [17] suggested that increased ordering of lipids of the viral envelope may contribute to improved stability at lower temperatures. There are several lines of evidence suggesting a relationship between the pH of haemagglutinin activation and stability of influenza viruses at higher temperatures; viruses with a pH of fusion of greater than 5.6 are more stable at 50°C compared with those with a pH of fusion less than 5.4 [18–23]. These studies demonstrate that temperature can impact influenza viruses at a molecular level.

We have previously reviewed the effect of humidity on survival of a broad range of viruses, including influenza virus [24]. Studies of influenza virus survival in droplets and aerosols as a function of RH have shown either a monotonic decrease in viability with increasing RH or a U-shaped relationship with reduced viability at mid-range RHs [25,26]. Disparities in results may be due to differences in the suspension media, whether it included protein or not [24].

When weighing the effect of humidity and limiting the variables under consideration to AH and RH only, and not temperature, one drawback is that high values of AH are only achievable at high temperature. Zhao *et al*. [27] recognized this in a study of survival of aerosolized Gumboro virus, concluding, ‘A large part of the AH effect on viral survival actually was attributable to the effect of temperature’. They found that temperature and RH together were the best predictors of virus survival and that survival was not significantly affected by AH. Because Gumboro virus is non-enveloped, its relationship between viability and RH is likely to differ from that of influenza virus [26], but the point about the relationship among temperature, AH and RH is universally applicable. It is well established that influenza virus is less stable at higher temperatures. Higher values of AH are only achievable at higher temperature, at which virus stability is compromised. Under this constraint, we might expect the correlation of viability with AH alone, which includes temperature by proxy, to be stronger than the correlation of viability with RH alone.

### 3.3. Reanalysis of virus survival data

Ideal linear regression models (i.e. linear in the coefficients) arise from uncorrelated independent variables. The functional form for AH in eqn (2.1) indicates that AH is dependent on *T*. Linear regression models with correlated independent variables often have inflated variances of the estimated coefficients, coefficients with signs in the opposite direction of known relationships and inaccurate predictions for dependent variable combinations inconsistent with the regressor correlation. Given this information, only two possible general linear models are statistically advised in this circumstance: either viability must be modelled as a function of RH and *T*, or it must be modelled as a function of AH. In general, when a known physical relationship indicates the presence of collinearity among regressors, there are two choices to fit the best-performing model: (i) fit a model with the less correlated or uncorrelated regressors or (ii) fit a model that accounts for collinear regressors (e.g. ridge regression, principal components regression and LASSO). The first, simpler approach is possible with this dataset.

**Figure 1.**
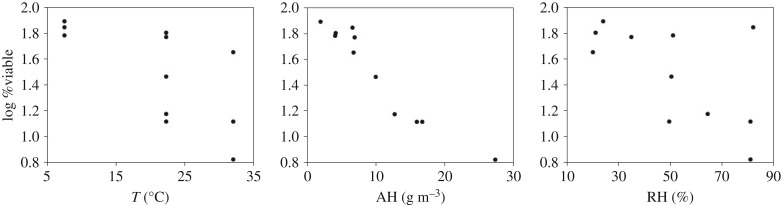
Scatterplots showing influenza virus viability in aerosols [13], quantified as the log of the per cent viable after 1 h, versus temperature (*T*), AH and RH.

Figure 1 shows scatterplots of Harper's original data [13] on influenza virus survival, where viability is quantified as the log of the percentage of infectious virus remaining after 1 h. Figure 1 indicates linear relationships of virus viability with the individual variables of RH and *T* and a nonlinear relationship with AH. Figure 1*a* shows a poor fit at higher temperatures, which can be explained by RH, as shown in a modified version of the figure in the electronic supplementary material, where viability is coloured by RH. At first glance, the relationship with AH shown in figure 1*b* appears to be linear; however, there is a slight curvature in the centre of the plot which is reflected in the predictive models described below. As shown in figure 1*c*, RH alone is a poor predictor, although it is evident that at low RH, viability is high. Over the range of values in the experiment, AH and *T* have a correlation coefficient of 0.651 (*p* < 0.0001).

A model containing *T*, RH and their interaction as predictors is comparable, in terms of *R*^2^, mean square error and predicted versus actual values, to a quadratic model containing only AH, as shown in table 1. Fitting the additional AH quadratic term results in a 15% reduction in the model root mean square error in comparison with the linear AH model, indicating a better fit to the data. Because AH is a function of both RH and *T*, any model that is a function of AH is effectively a function of RH and *T*. The electronic supplementary material contains parameter estimates for the models. While it may appear to be more appropriate to compare a model containing *T*, RH, *T* × RH with a model containing *T*, AH, *T* × AH, this is not the best approach for the Harper data. The correlation between *T* and AH (*ρ* = 0.651) in this specific dataset results in statistical models indicating no relationship between viability and *T*, contrary to scientific knowledge. In addition, the root mean square error increases by 20% in the *T*, AH, T × AH model. This is a classic manifestation of multicollinearity (correlated regressors) in a regression model and is the reason this model is not presented for discussion. We fitted multiple additional models to the data, but do not include them in the discussion because they either contain correlated regressors or do not have the same predictive capability as the chosen models.

**Table 1.** Measures of goodness of fit comparing models of airborne virus viability [13] as a function of RH and *T* and as a function of AH only.

measure of fit	*T*, RH, *T* × RH	AH, AH × AH
*R*^2^	94.8%	94.3%
mean square error	0.10	0.10

**Figure 2.**
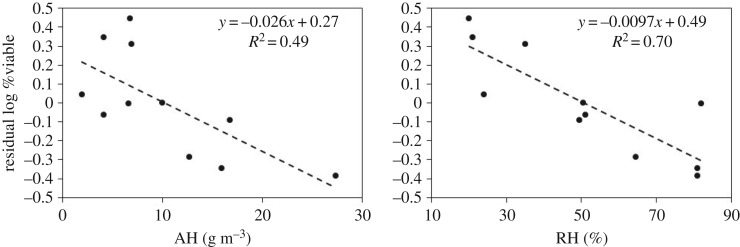
Residuals of influenza virus viability, quantified as the log of the per cent viable after 1 h, versus AH and RH after removing the temperature effect.

Another way of showing the influence of both temperature and humidity on virus viability is to first remove the temperature dependence and then examine the relationship between humidity and the residuals. Figure 2 shows the residuals of the linear regression of virus viability against temperature as a function of AH and RH. In both cases, the slope is negative, but the goodness of fit is better with RH than with AH (*R*^2^ of 0.70 versus 0.49).

In summary, the *T* and RH linear model with interactions and the quadratic AH model perform similarly in their ability to explain and predict influenza virus viability for this experimental dataset. The statistical models agree with the fundamental physical laws and equations that govern the relationships among the three quantities of *T*, AH and RH. For statistical purposes, we can interchange the two because a model based on AH is a function of *T* and RH and vice versa. First- and second-order models approximate relationships well in local areas. However, as the ranges of the independent variables change, the values of the coefficients, the order of the model and/or the significant terms are subject to change in order to obtain the best approximation [28].

At first glance, this result appears to conflict with previous studies concluding that AH modulates influenza virus survival, transmission and seasonality [1–4]. However, closer inspection shows that our result is consistent with these studies. While Shaman & Kohn's [3] analysis of Harper's data used simple linear regression with only one variable at a time and did not account for an increase in the variance of the response, we fit a more complex model that was appropriate given multicollinearity in the dataset. Had we employed Shaman & Kohn's [3] approach, we would have obtained the same results as they did. Using an epidemiological model, Shaman *et al*. [4] subsequently showed that AH alone could explain influenza seasonality in the USA. They demonstrated a statistically stronger association of AH than RH with the onset of wintertime influenza on the basis of comparing *p*-values of less than 0.00002 for AH and 0.00166 or lower for RH (table 1 in [4]), but small differences in *p*-values should not be used to rule out the influence of one variable versus another. McDevitt *et al*. [2] studied the inactivation of influenza virus on stainless steel surfaces and found that ‘The multiple linear regression model including independent variables for RH, temperature, and time…provides a reasonable means of predicting virus survival, but it does so at the expense of adding an additional parameter to the model’, compared with the model including only AH and time. There was very little practical difference in the adjusted *R*^2^ values of the two models (0.90 versus 0.93, respectively), as in our results (table 1). Although the authors selected the model with AH and time because it was simpler, this choice does not invalidate the strength of the model containing RH, temperature and time. In an analysis of global observational data, Deyle *et al*. [1] stated that the effect of RH on influenza incidence was weaker than that of AH or temperature, but it was not absent. The magnitude of the weakness was not indicated, and the authors did not consider a model with the interaction term *T* × RH, which might have produced different results.

We recently conducted a study that was explicitly designed to discriminate between AH and RH in the survival of the bacteriophage phi6, which has been suggested as a surrogate for influenza virus [29], in aerosols and droplets. The application of linear and log-linear multiple regression models to the data produced modest fits and varying results about the significance of AH and RH, but we achieved the best fit to the results using a random forest model, which indicated that RH and temperature were stronger predictors of virus survival than was AH [30].

We caution against using these results to draw conclusions about virus survival under real-world conditions. Influenza virus morphology can range from spherical, as in the laboratory-adapted strain A/Puerto Rico/8/34 (PR8), to filamentous, as maintained in nature [31], and the effect of morphology on virus survival is not known. Numerous studies have shown that the relationship between virus survival and humidity depends on the chemical composition of the suspension media. Both salt and protein concentrations affect virus viability [25]. We have recently shown that in a physiologically realistic suspension medium, specifically extracellular matrix from human bronchial epithelial cells, the strain A/California/07/2009 (H1N1pdm09) survives well in both droplets and aerosols independent of RH [32]. More studies are needed to confirm the relationship between strain, morphological phenotype, temperature, humidity and influenza virus survival in real respiratory fluid.

### 3.4. Influenza transmission in animal models

Limited animal studies have been performed to elucidate the role of humidity on the transmission of influenza viruses. Studies in guinea pigs [33] and ferrets [34] have shown that dry conditions favour transmission. Reanalysis of the guinea pig results suggested that AH, rather than RH, modulates transmission [3], although the authors of the guinea pig study [35] subsequently noted, ‘several of our transmission results are not explained by this relationship’. Transmission was completely blocked at 30°C and at two different fixed values of AH, transmission efficiency spanned the full range from 0 to 100% of contacts infected. Analysis of the transport of the virus between cages inside the chamber suggested that viral growth kinetics and background airflow in the chamber could explain the observations without invoking virus survival [36]. In addition, given the rapid forced airflow rates within experimental animal transmission systems, an estimated 10 s would have elapsed between release of the virus from the infected subjects and exposure to the test subjects [36]. This amount of time is likely to be insufficient for environmental conditions to affect the viability of viruses in released aerosols.

A carefully designed study is needed to test the hypothesis that humidity affects transmission by modulating virus survival. To isolate the effect, researchers would need to collect viral aerosols from infected animals and keep them suspended to allow sufficient time for ageing and inactivation at different humidities before exposing uninfected animals, ideally using a method that isolated transmission by the aerosol mode. Simultaneous measurements of virus loading in air, both in terms of total genome copies and infectious virus, are advisable to control for varying amounts of virus released by infected animals.

If the results from animal model influenza transmission studies are to be extrapolated to humans, greater understanding is needed of how the anatomical and symptomatic differences between animal models and humans contribute to the observed transmission characteristics [37,38]. Ferrets have been considered the ‘gold standard’ for influenza transmission studies, but there are several key physical differences between ferrets and humans that may impact how any transmission patterns (at different temperatures and humidities) seen in ferrets can be extrapolated to humans due to various allometric scaling issues. Some of these differences include body posture and the direction in which nostrils point; diameter, length and morphology of the airways; airflow velocity; respiratory rate and body temperature. All of these variables may affect the dynamics and deposition of inhaled particles of different sizes at different temperatures and humidities.

### 3.5. Epidemiology

In studies of influenza incidence versus meteorological conditions, some have found a stronger relationship with AH than RH, while others have reached the opposite conclusion. Davis *et al*. [39] reported an association between cold, dry conditions and influenza mortality in New York City. Using causality analysis on a global flu data, Deyle *et al*. [1] showed that AH, and to a lesser extent temperature, drives influenza incidence. Tang *et al*. [40] documented that the incidence of influenza A in Hong Kong increased with higher RH, while the incidence of influenza B decreased with a higher temperature and the incidence of neither was related to AH. In a study of paediatric hospital admissions in Singapore, Loh *et al*. [41] found that upper respiratory tract infections, which were correlated with positive test results for influenza A, increased with lower RH and were not related to AH. Some of the apparent disagreement in results may be due to differences in climate. On average, Hong Kong and Singapore are warmer and more humid than New York City, and air conditioning and heating practices, which can dramatically influence indoor humidity, are likely to differ among locations. In some cases, the association with one measure of humidity versus another is weaker but not absent.

Because people spend over 90% of their time indoors [42], it is likely that influenza transmission among humans occurs mainly indoors rather than outdoors. If so, then indoor, rather than outdoor, environmental conditions should be more closely related to transmission, but indoor data are not widely available, especially for humidity. Nearly all of the epidemiological studies investigating links between temperature, humidity and influenza incidence have relied on outdoor measures of temperature and humidity. One exception is a study in a low-resource tropical setting with a limited sample size of 34 households [43]; the study did not detect differences in transmission efficiency as a function of temperature or humidity. To aid interpretation of epidemiological studies that use outdoor environmental data, we must understand the relationship between outdoor and indoor conditions.

Despite its seeming simplicity, or perhaps because of it, there have been few studies of the relationship between indoor and outdoor temperature and humidity. Indoor and outdoor temperatures were associated during the warm season but not during the cool season in New York City [44]. Indoor temperature measured in eight locations ranging from near the equator to the Arctic showed little variation across location and season [45]. A study in Boston, Massachusetts, showed that indoor and outdoor temperatures were strongly correlated when outdoor temperatures were greater than 13°C but only weakly correlated when outdoor temperatures were lower [46]. In all three studies, outdoor RH was only weakly correlated with indoor RH, while outdoor AH was strongly correlated with both indoor AH and indoor RH [44–46]. Unless water vapour is removed by an air conditioning unit or dehumidifier, or added by a humidifier, we expect indoor AH to be similar to that outdoor AH because of air exchange in a building, although sources such as humans, vegetation and water use could contribute water vapour to indoor air while absorptive materials could remove it. Indoor AH and RH are expected to be strongly correlated because indoor temperature is usually controlled to within a fairly narrow range [47], especially during winter unless the heating system employs humidification. Because AH and RH are proportional and move in tandem at a fixed temperature, it would be difficult to identify one versus the other as having a more significant effect indoors. We agree with Nguyen & Dockery [45] that outdoor AH serves a reliable proxy for indoor AH and RH in temperate regions, while outdoor RH does not.

In temperate regions during the cold season, indoor air is usually heated, and indoor and outdoor environmental parameters differ significantly. The cold season typically coincides with the peaks of influenza incidence in these regions. Although some studies have speculated that influenza seasonality may be related to indoor crowding effects [48,49], a causal relationship has not been shown [50]. There is now a need to study the relationship between these seasonal winter influenza incidence peaks and indoor, rather than outdoor, environmental parameters. In studies showing that influenza seasonality is linked to outdoor AH in temperate regions, it is likely that outdoor AH is serving as a proxy for indoor AH or RH.

In tropical regions, the relationship between indoor humidity and outdoor conditions is not as easily predicted. Air conditioning removes water vapour from air, but the resulting indoor humidity will depend on the temperature differential between air entering and leaving the unit and the fraction of air that is recirculated. The prevalence of air conditioning varies with income and wealth [51], and thus, there may be differences in the indoor–outdoor humidity relationship as a function of these factors. One study found lower values of temperature and RH in businesses that needed to appear ‘high-status’, such as banks, dining facilities, retail shops and offices, compared with other buildings in tropical countries [52]; the researchers suggested that such conditions were more favourable for aerosol transmission of influenza. In a small study of influenza transmission in tropical settings that found no differences in transmission efficiency with indoor temperature or humidity, Tamerius *et al*. [43] concluded, ‘observational studies investigating the relationship between environment and influenza activity should use caution using outdoor environmental measurements since they can be imprecise estimates of the conditions that mediate transmission indoors’.

### 3.6. Mechanisms

When influenza viruses are emitted into air from an infected host by coughing, sneezing, talking or breathing [53–55], they are just one component of respiratory droplets that are a complex mixture of inorganic and organic ions, protein and surfactant [56]. The respiratory droplets can range in size from submicrometre to thousands of micrometres [57–64], although those larger than 100 µm remain suspended for less than 5 s before settling to the ground from a height of 1.5 m. We expect temperature to affect the stability of viruses in the environment, including those that are airborne. In a review of the effect of environmental parameters on the survival of airborne pathogens, Tang [26] concluded that temperature and RH always interact to affect the survival of aerosolized viruses. One mystery is how RH could affect viruses in a respiratory droplet because unless they are present at the surface of the droplet, they are not exposed to ambient air and so would not directly interact with water vapour in the air.

Upon contact with ambient air, respiratory droplets are subject to evaporation until they reach equilibrium. Evaporation may be partial, in which case some water remains in liquid or semi-solid form, or complete, in which case only solutes and the virus remain, and likely some trapped water molecules. Air expired from the respiratory tract is saturated [65], whereas ambient air usually is not, so there is a driving force in the form of a vapour pressure gradient between the surface of a droplet and ambient air. The extent of evaporation and final, equilibrium size of the droplet is determined by ambient RH, not AH. This equilibrium size, which is attained within a few seconds at most [3,36,66], has important effects on the droplet's physics and chemistry, although inactivation of any virus contained in the droplet appears to proceed more slowly [13].

**Figure 3.**
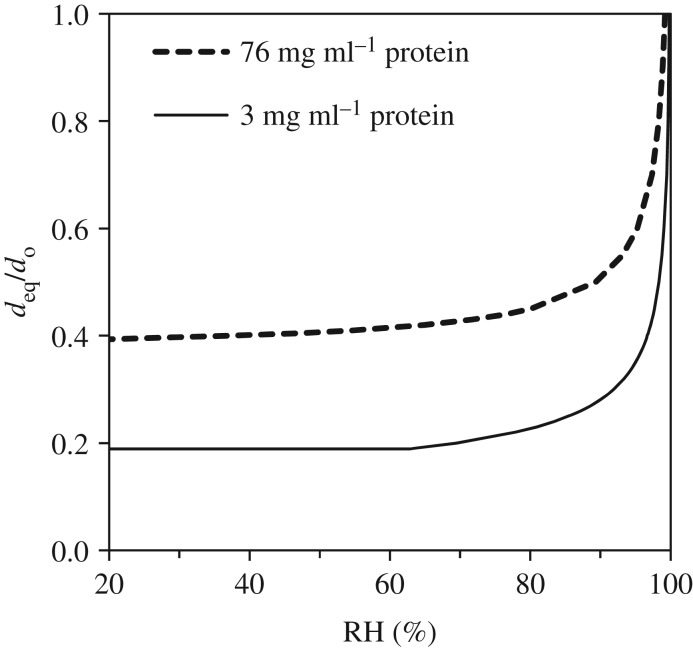
Ratio of equilibrium diameter to initial diameter (*d*_eq_/*d*_o_) as a function of RH for a droplet consisting of 9 mg ml^−1^ NaCl, 3 or 76 mg ml^−1^ protein and 0.5 mg l^−1^ surfactant.

Combining the effects of curvature at the air–liquid interface (i.e. the Kelvin effect) and of solutes on the saturation vapour pressure of a droplet produces an equation that can be used to predict the equilibrium diameter of a droplet as a function of RH [67]. Figure 3 shows how the equilibrium size of a droplet of model respiratory fluid depends on RH in terms of the ratio of the equilibrium diameter to the initial diameter. Initially, the model fluid contains 9 mg ml^−1^ salt as NaCl, 3 or 76 mg ml^−1^ protein (range of values found in nasal surface airway fluid [10] and aerosols in exhaled breath condensate [11], respectively) and 0.5 mg ml^−1^ surfactant as DPPC [12]. For reference, two other studies report total protein contents of respiratory fluid that are closer to the lower end of the range: 4 mg ml^−1^ in unstimulated saliva [68] and 7 mg ml^−1^ in nasal mucus [69].

The ratio shown in figure 3 applies for droplets of initial diameter as small as approximately 0.5 µm, at which point the Kelvin effect becomes important. Figure 3 shows that at 100% RH, there is no evaporation, and the ratio is 1. At 90% RH, the droplets are dramatically smaller at equilibrium, 0.28 or 0.51 of their initial size for low and high-protein contents (3 mg ml^−1^ and 76 mg ml^−1^), respectively. At 50% RH, these values are 0.19 and 0.41, respectively. For comparison, Liu *et al*. [70] predicted that dried droplet nuclei would be 0.32 of their original diameter. In fact, at RH below 64% for the low-protein droplets and below 42% for the high-protein droplets, the equilibrium diameter is unchanged because all bulk liquid water has been lost and only the solutes, or what some have called the droplet nucleus, remain.

The equilibrium droplet size is critical in determining its fate (e.g. [71–74]). Size determines how long the droplet remains suspended in air before it is removed by gravitational settling and where it deposits in the respiratory system if inhaled. The transformation of a droplet subject to evaporation in ambient air has a dramatic impact on its lifetime. We calculated the settling time for a droplet initially 10 µm in diameter containing 9 mg ml^−1^ salt as NaCl and 3 mg ml^−1^ protein at RHs of 100%, 90% and 64% and lower. Table 2 shows that the droplet shrinks to 2.8 µm at 90% RH and to 1.9 µm at less than 64% RH. These diameters correspond to vastly different settling times; the 10 µm droplet at 100% RH remains suspended for only 8 min, whereas the 1.9 µm droplet at less than 64% RH remains suspended for more than 3 h, posing a much longer opportunity for airborne transmission. Xie *et al*. [74] and Parienta *et al*. [72] present a much more complete analysis of droplet transformation and transport distance as a function of initial size and RH.

**Table 2.** Parameters for a droplet initially 10 µm in size containing 9 mg ml^−1^ NaCl, 3 mg ml^−1^ protein and 0.5 mg ml^−1^ surfactant.

	100% RH	90% RH	<64% RH
equilibrium size	10 µm	2.8 µm	1.9 µm
time to settle 1.5 m	8 min	102 min	216 min
deposition efficiency in head airways	81%	73%	57%
deposition efficiency in tracheobronchial region	2%	6%	6%
deposition efficiency in alveolar region	2%	10%	12%
concentration factor	1	65	>7000

Furthermore, we can calculate the deposition efficiency in different regions of the respiratory system to contrast where the droplets might deposit if inhaled [75]. Table 2 shows that the 10 µm droplet has a high chance of being deposited in the head airways (81%) and only a small chance of being deposited in the alveolar region (2%), whereas a 1.9 µm droplet has a lower chance of being deposited in the head airways (57%) and a relatively higher chance of being deposited in the alveolar region (12%) than does the 10 µm droplet. This calculation does not account for hygroscopic growth of droplets upon entering the saturated airway, although we have shown that model respiratory droplets containing surfactant do not reabsorb water even after extended exposure to saturated conditions [56]. A review of aerosol transmission of influenza suggests that infection initiated in the lower respiratory tract requires a lower dose and produces more severe symptoms compared to intranasal inoculation [76]. In summary, at lower RH, droplets stay suspended longer and are more likely to deposit in the lower respiratory tract. As mentioned earlier, the dynamics and deposition efficiencies of aerosols in animal models are likely to be quite different, for example in ferrets due to their more horizontal posture and much smaller airway diameters compared to those of humans [37,38].

Besides its effects on physics of a droplet, evaporation also affects its chemistry, which could affect the stability of a virus contained within the droplet. The mass of solutes in a respiratory fluid droplet containing one virus is expected to be at least five orders of magnitude larger than the mass of the virus [56], so these components should not be ignored when considering virus viability in droplets and aerosols. Table 2 shows the concentration factor, calculated as the ratio of the initial mass of water in the droplet to that remaining at equilibrium. This is the factor by which solutes that remain in the aqueous phase become concentrated due to loss of water if they do not precipitate out of solution. The solubility of NaCl is 360 mg ml^−1^, or 40 times the initial concentration of 9 mg ml^−1^. Thus, we expect it to crystallize at an ambient RH of approximately 90% (concentration factor of 65) and lower, and indeed, we have observed crystallization of salt in model respiratory fluid droplets exposed to varying RH [25,56]. Under these concentrated conditions in droplets, protein may form aggregates [56], and there may be spatial gradients in pH [77]. In studies of atmospheric aerosols, researchers have observed phase separation, crystallization and changes in pH under conditions of changing RH [78–81]. While such changes in droplet chemistry could very well cause virus decay, the exact mechanism of inactivation is not known. Various possibilities have been proposed, such as osmotic bursting [82] or pH-sensitive changes in protein structure [24], but more studies are needed.

## 4. Conclusion

The debate over the importance of AH versus RH for influenza virus survival and transmission dates to the mid-twentieth century. Writing in *Nature* in 1960, Hemmes *et al.* [83] claimed, ‘it became evident that the relative humidity and not the absolute humidity is the determining factor’, for virus survival in aerosols. Analyses of the early twenty-first century have pointed to AH as the modulating factor. While it is appealing to try to isolate a single controlling environmental factor that modulates influenza virus survival, transmission and incidence, our analysis suggests that the combination of temperature and RH provides a consistent, mechanistically sound explanation of the observations. Temperature can be considered an intrinsic factor in virus stability because rates of inactivation of proteins and nucleic acid are expected to increase with temperature [16]. RH can be considered an extrinsic factor in virus stability because it controls evaporation, which affects a droplet's size and physical fate and its chemical microenvironment. By contrast, there is no mechanism by which AH is expected to affect droplet diameter except through its relationship with RH.

The story is not yet complete, but we know that RH determines the extent of evaporation of an airborne droplet and thus the resulting chemical microenvironment to which the virus is exposed. If virus survival in the environment is a dominant factor in influenza transmission, then RH is expected to influence incidence and seasonality, too.

## Supplementary Material

Derivation of humidity equation

## Supplementary Material

Virus viability colored by temperature

## Supplementary Material

Parameter estimates for model fits

## Data accessibility

All data used in this work have been previously published and can be found in the cited references.
