# Supplementary material for: Mechanistic insights into the effect of humidity on airborne influenza virus survival, transmission and incidence
Source: J R Soc Interface. 2019 Jan 16;16(150):20180298. doi: 10.1098/rsif.2018.0298 (PMC6364647; doi:10.1098/rsif.2018.0298)
Supplement: Derivation of humidity equation [file rsif20180298supp1.docx]

Supplementary Material

Mechanistic insight into the effect of temperature and humidity on airborne influenza virus survival, transmission, and incidence

Linsey Marr, Julian Tang, Jennifer Van Mullekom, Seema Lakdawala

**Equation for absolute humidity (AH) as a function of relative humidity (RH) and temperature (T)**

An improved form of the Magnus equation for the saturation vapor pressure of water is

$$P_{sat}=610.94 exp\left( \frac{17.625 T}{T+243.04} \right)$$

where *P_sat_* is the saturation vapor pressure in Pascals (Pa) and T is temperature in °C, valid over the range of -40 °C to 50 °C [1]. If we combine this with the ideal gas law to convert partial pressure to a mass concentration, we obtain the relationship

$$C_{sat}=\frac{610.94 exp\left( \frac{17.625 T}{T+243.04} \right)\times18}{8.314\left( T+273.15 \right)}$$

where C_sat_ is the concentration of water vapor under saturated conditions in g m^-3^, 8.314 J mol^-1^ K^-1^ is the ideal gas constant, and 18 g mol^-1^ is the molecular mass of water. Finally, we can combine constants and multiply by RH to obtain an empirical equation for AH in terms of RH and T:

$$AH=\frac{1322.7exp\left( \frac{17.625 T}{T+243.04} \right)\times\left( \frac{RH}{100} \right)}{T+273.15}$$

where AH is in g m^-3^ and RH is in percent.

**References**

[1] Alduchov OA, Eskridge, RE. 1996 Improved Magnus form approximation of saturation vapor pressure. *J. Appl. Meteorol.* **35**, 601-609. (doi:10.1175/1520-0450(1996)035<0601:Imfaos>2.0.Co;2)
