# Supplementary material for: Mechanistic insights into the effect of humidity on airborne influenza virus survival, transmission and incidence
Source: J R Soc Interface. 2019 Jan 16;16(150):20180298. doi: 10.1098/rsif.2018.0298 (PMC6364647; doi:10.1098/rsif.2018.0298)
Supplement: Virus viability colored by temperature [file rsif20180298supp2.docx]

Supplementary Material

Mechanistic insight into the effect of temperature and humidity on airborne influenza virus survival, transmission, and incidence

Linsey Marr, Julian Tang, Jennifer Van Mullekom, Seema Lakdawala

| 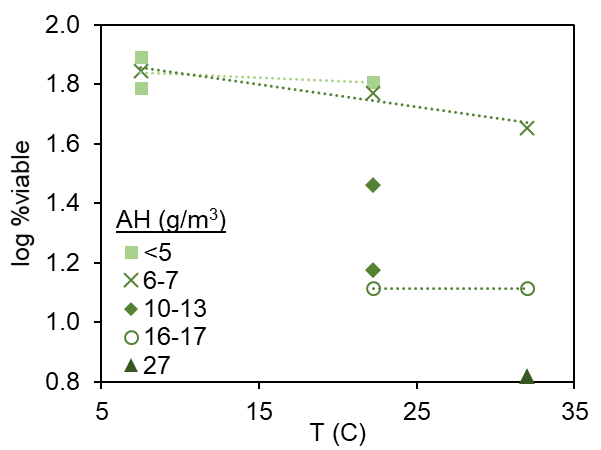 | 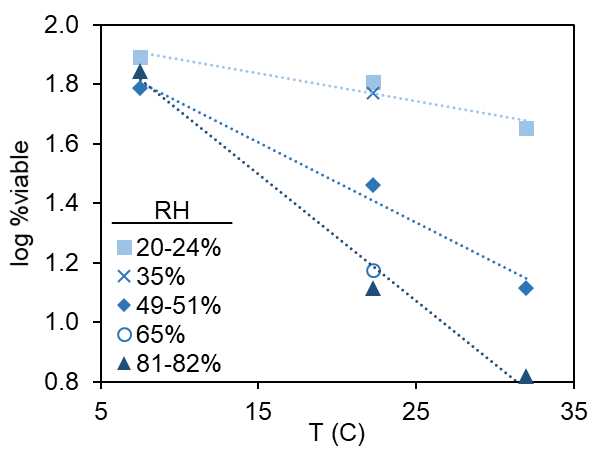 |
| --- | --- |
| 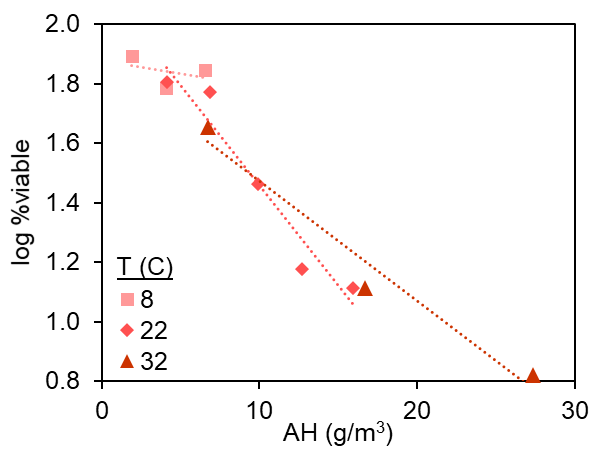 | 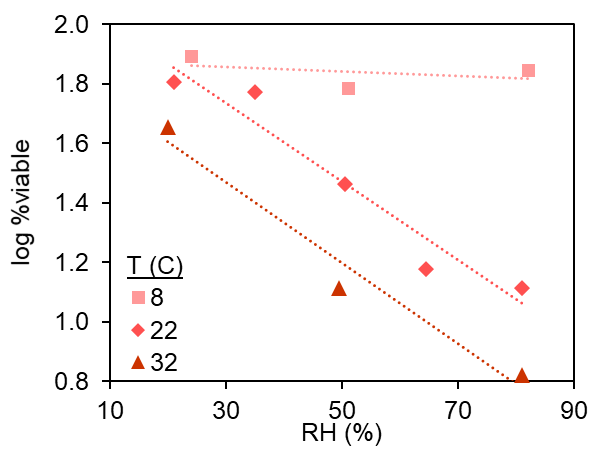 |

**Figure S1.** Scatterplots showing influenza virus viability in aerosols [13], quantified as the log of the percent viable after 1 hr vs. temperature (T), colored by absolute humidity (AH) or relative humidity (RH); AH colored by T; and RH colored by T. Trendlines are also shown.
