# Supplementary material for: Mechanistic insights into the effect of humidity on airborne influenza virus survival, transmission and incidence
Source: J R Soc Interface. 2019 Jan 16;16(150):20180298. doi: 10.1098/rsif.2018.0298 (PMC6364647; doi:10.1098/rsif.2018.0298)
Supplement: Parameter estimates for model fits [file rsif20180298supp3.docx]

Supplementary Material

Mechanistic insight into the effect of temperature and humidity on airborne influenza virus survival, transmission, and incidence

Linsey Marr, Julian Tang, Jennifer Van Mullekom, Seema Lakdawala

**Parameter estimates for model of influenza virus viability in aerosols as a function of temperature (T) and relative humidity (RH) and of absolute humidity (AH)**

These estimates stem from statistical models using ordinary least squares regression with normal error structure to fit log % viability as a function of T, RH, and AH.

Model involving T and RH

| **Term** | **Estimate** | **Std Error** | **t Ratio** | **Prob>\|t\|** |
| --- | --- | --- | --- | --- |
| Intercept | 2.5413675 | 0.105708 | 24.04 | <.0001* |
| T (C) | -0.027002 | 0.003409 | -7.92 | <.0001* |
| RH (%) | -0.009577 | 0.001357 | -7.06 | 0.0002* |
| (T (C)-20.8864)*(RH (%)-50.8636) | -0.000551 | 0.000141 | -3.91 | 0.0058* |

Model involving AH only

| **Term** | **Estimate** | **Std Error** | **t Ratio** | **Prob>\|t\|** |
| --- | --- | --- | --- | --- |
| Intercept | 2.0226936 | 0.055654 | 36.34 | <.0001* |
| AH (g/m3) | -0.056946 | 0.005949 | -9.57 | <.0001* |
| ( AH (g/m3)-10.2674)*( AH (g/m3)-10.2674) | 0.0011221 | 0.000537 | 2.09 | 0.0701 |
